# Injury-Induced Remodeling of Junctional Actin Bands in the Vestibular Maculae of Mice and Chicks: Implications for Sensory Regeneration

**DOI:** 10.1007/s10162-026-01040-4

**Published:** 2026-03-03

**Authors:** Mark E. Warchol

**Affiliations:** Department of Otolaryngology, Washington University School of Medicine, 4523 Clayton Ave., St. Louis MO, 63110 MO USA

**Keywords:** Ototoxicity, Regeneration, YAP signaling, Cytoskeleton, Hair cell, Supporting cell

## Abstract

The vestibular organs of birds are capable of regenerating sensory hair cells after ototoxic injury, but the regenerative ability of the mammalian vestibular organs is much more limited. The factors that inhibit regeneration in the mammalian inner ear are not known, but it has been proposed that the structure of filamentous actin cables at cell–cell junctions may be an important regulatory influence. Junctional actin cables in the chick utricle are relatively thin, while those in the mouse utricle are much thicker. These differences result in differing mechanical properties of the avian vs. mammalian inner ear, which may affect the potential for regenerative proliferation. In the present study, I characterized injury-evoked changes in junctional actin cables and supporting cell surface areas in the utricles of mice and chicks of either sex. I found that the thickness of junctional cables in the chick utricle was not affected by ototoxic injury, but that injury to the mouse utricle resulted in the formation of numerous new junctional actin bands whose thickness was comparable to those in the chick utricle. Thicker actin bands also persisted after injury, but were not necessarily associated with cellular junctions. I further found that moderate hair cell injury caused supporting cells in the chick utricle to expand their lumenal surfaces by about 50%, while comparable injury to the mouse utricle caused supporting cells to expand by only ~ 30%. I speculate that this difference may impact the injury-induced activation of Hippo/YAP signaling.

## Introduction

The sensory hair cells of the vestibular organs detect head movements and the orientation of the head with respect to gravity. Vestibular hair cells can be damaged or lost following ototoxicity or as a consequence of normal aging. Nonmammalian vertebrates can regenerate vestibular hair cells after ototoxic injury, leading to functional recovery. This process of hair cell regeneration has been particularly well-studied in the inner ears of birds and occurs largely (but not exclusively) by the renewed proliferation of supporting cells, which divide to produce new hair cells and supporting cells [1]. In contrast, the vestibular organs of mammals have a very limited ability to regenerate hair cells [2]. Mammalian vestibular organs can produce a moderate number of new hair cells after severe injury, but those replacement cells do not fully differentiate and do not restore sensory function [3, 4]. Ototoxic damage to the mammalian vestibular organs also results in relatively low levels of supporting cell proliferation [5, 6]. Together, these observations suggest a link between the ability of supporting cells to divide in response to hair cell injury and the potential for functional hair cell regeneration.

Most studies of vestibular regeneration in birds and mammals have focused on the utricle, a sensory organ that detects linear acceleration. The factors that are permissive for regenerative proliferation in the avian utricle and that limit such proliferation in the mammalian utricle are not fully understood, but recent work has suggested a key role for mechanical signals in regulating cell cycle entry. Cell–cell junctions in all vestibular epithelia possess circumferential actin cables, which link a cell’s cytoskeleton to the adherins junctions between adjoining cells. In the vestibular organs of birds, these actin bands are relatively thin (~ 0.5 µm), while those in mammals are considerably wider (~ 3 µm) [7]. Junctional actin bands in utricles of neonatal mice are thin and gradually become thicker during subsequent maturation. Notably, this increase in thickness is correlated with reduced proliferative ability [7–9]. These observations suggest that the structure of junctional actin bands and the mechanical environment of the sensory epithelium may regulate the potential for regenerative proliferation.

The lumenal surfaces of supporting cells also undergo rapid expansion after hair cell loss [10], and it is possible that this cellular expansion may trigger renewed proliferation. Mechanical regulation of cell division is mediated by the YAP signaling pathway, which can be activated by cellular stretching [11]. Prior studies have shown that YAP signaling regulates proliferation in the developing and regenerating utricle [12–14], but the relative degree of supporting cell expansion in birds vs. mammals has not been quantified. To better understand the mechanical factors that may influence regeneration in the utricle, the present study quantified changes in the width of circumferential actin belts and the spreading of supporting cells in the utricles of chicks and mice after ototoxic injury. Results indicate that injury to the chick utricle did not affect the thickness of circumferential actin bands, but that hair cell loss in the mouse utricle led to the formation of numerous thin actin bands. In addition, hair cell loss caused a greater relative degree of cellular expansion in the chick utricle than in the mouse utricle. These results may have implications for the differential activation of YAP signaling in the vestibular organs of these species.

## Methods

### Animals

Studies used mice of both sexes on a 129S1/SvImJ:C57Bl/6 J background (JAX #101045). Chickens (White Leghorn strain) were hatched from fertile eggs (Charles River SPAFAS) and maintained in heated brooders. Chicks and mice had ad libitum access to food and water and were housed in the animal facilities of Washington University. All protocols involving animals were approved by Institutional Animal Care and Use Committee (IACUC) of Washington University, School of Medicine, in Saint Louis, MO.

### Ototoxic Lesions

Mice (age 2–4 months) received a single i.p. injection of 3,3'-iminodipropanenitrile (IDPN, TCI America, mixed 1:1 in sterile PBS) at a dose of 4 mg/gm. Mice were maintained for an additional 3–56 days after IDPN injections. Chickens (2–4 weeks post-hatch) received injections of streptomycin sulfate (1200 mg/kg, i.m.) once/day for three consecutive days and were then allowed to recover for 24 h after the last injection. All animals were euthanized by CO_2_ inhalation and isolated temporal bones (mice) or utricles (chicks) were immediately removed and fixed in for 1 h 4% paraformaldehyde in 0.1 M phosphate buffer.

### Immunohistochemistry

After fixation, utricles were thoroughly rinsed in 0.01 M phosphate buffered saline (PBS, Sigma) and processed for immunohistochemistry as wholemounts. Specimens were first incubated for 2 h in PBS with 5% normal horse serum and 0.2% Triton X-100 (Sigma). They were then incubated overnight (at room temperature) in primary antibody solution. The following primary antibodies were used: anti-Myosin-VIIa antibody (rabbit polyclonal, Proteus BioSciences, 1:500), and anti-β-catenin (mouse monoclonal, BD Biosciences, 1:100). All antibodies were diluted in PBS with 2% horse serum and 0.2% Triton X-100). The next day, specimens were rinsed 3X (> 5 min each) in PBS and incubated for 2 h in secondary antibodies (conjugated to Alexa-488, Alexa-568, or Alexa 647, Life Technologies, 1:500 in PBS with 0.2% Triton X-100) at room temperature. Filamentous actin was labeled with Alexa Fluor 555-conjugated Phalloidin (Invitrogen), and cell nuclei were labeled with DAPI (Sigma, 1 μg/ml). Following histochemical processing, all samples were rinsed 3 × (> 5 min each) in PBS and mounted in glycerol:PBS (9:1) on glass slides.

### Imaging and Quantification

Specimens were imaged using a Zeiss LSM700 confocal microscope. Images were processed and analyzed using Volocity or ImageJ/Fiji Software. All quantification was conducted from high magnification images that were obtained using a 63 × 1.4 NA objective. Hair cell counts were obtained from four 2,500 µm^2^ regions (two in the striola, two in the extrastriola). ImageJ/Fiji software was used to quantify junctional thickness from phalloidin-stained specimens. Actin cable thickness between adjoining supporting cells was measured, beginning at the left side of a high magnification image and moving rightward (see tracings in Fig. 2A-C). The thickness of junctions between supporting cells and hair cells was not measured. Figures were assembled using Adobe Illustrator.

### Statistical Analysis

Data analysis and statistics were carried out using GraphPad Prism version 6.0d. Data are presented as mean ± SD. Student’s t-tests or analyses of variance (ANOVA) followed by Tukey’s or Bonferroni’s post hoc tests were applied, as appropriate.

## Results

### Remodeling of Junctional Actin Bands after Ototoxic Injury

Cellular junctions in the sensory epithelia of the inner ear possess filamentous actin bands that surround the inner perimeter of each cell and are linked to actin bands in adjoining cells via adherins junctions [8]. The thickness of these actin bands varies among the ears of different vertebrates and quantification of actin band thickness in vestibular maculae from five vertebrate classes suggests that the thick junctional actin cables in the mammalian ear may limit its regenerative abilities [8, 15]. Motivated by the study of Burns et al. [7], I first used phalloidin to label filamentous actin in the utricles of mice and chicks. The mean thickness of junctional bands in the mouse utricle was 2.88 ± 0.86 µm (n = 279 samples from seven specimens, ages: P28-52), while the mean thickness of junctional bands in the chick utricle was 0.40 ± 0.08 µm, n = 239 samples from five specimens, ages:10–14 days post-hatch). No differences in actin band thickness between the striolar and extrastriolar regions were noted in either species. These data indicate that junctional actin bands in the mouse utricle are ~ 6–7 × wider than those in the chick utricle and are in agreement with the observations of Burns et al. [7].

**Fig. 1:**
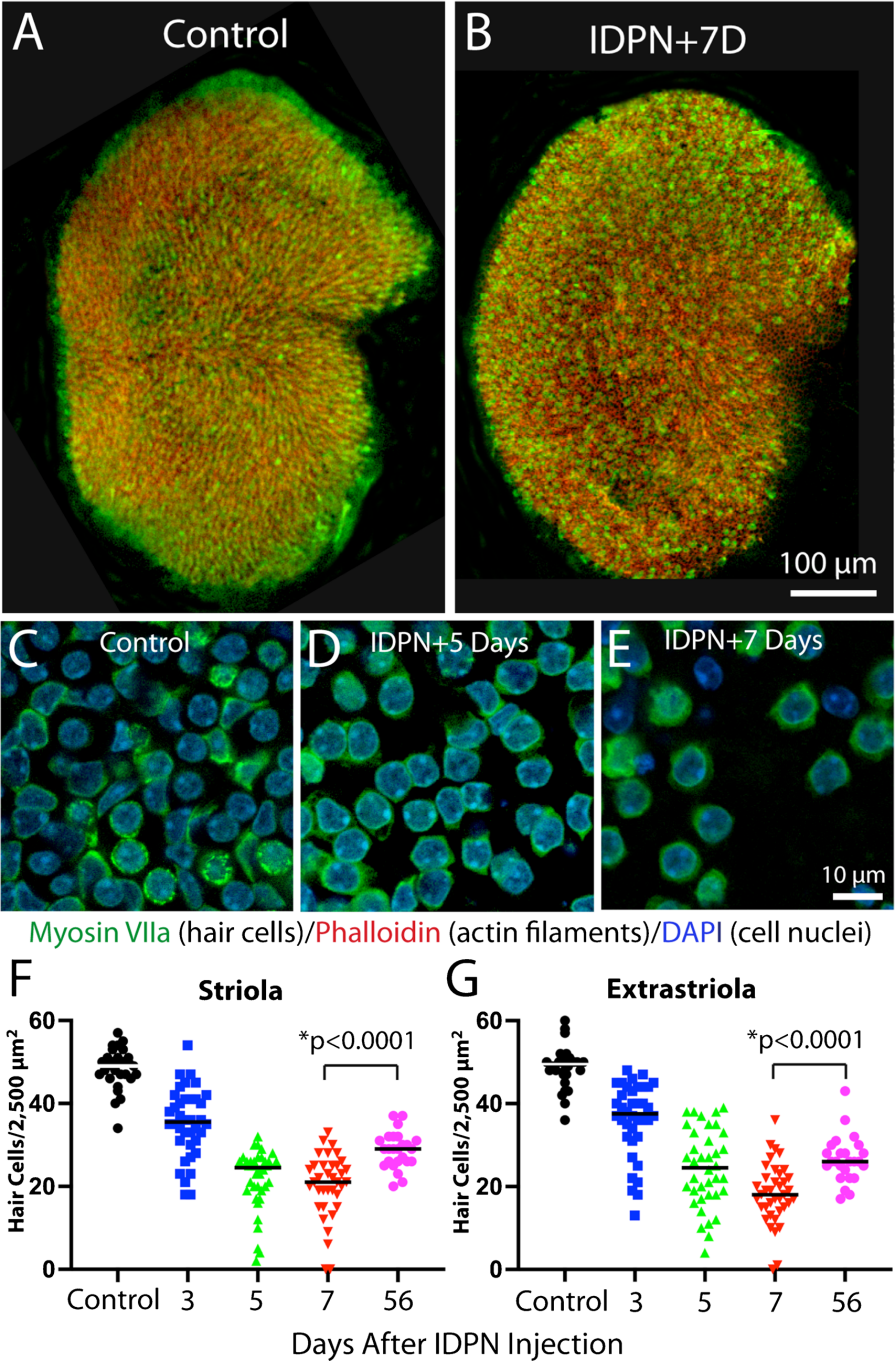
Ototoxic injury to the mouse utricle following systemic treatment with IDPN. Mice received a single 4 mg/gm injection of IDPN and were allowed to recover for 3–56 days. (**A**, **B**): At seven days post-IDPN, reduced hair cell density was observed in low magnification images of whole mounts. (**C**, **D**, **E**): Higher magnification images show hair cell loss. Such images were used to quantify hair cell density at various recovery times. (**F**, **G**): IDPN injection led to a progressive decrease in hair cell density in both the striolar and extrastriolar regions of the utricle. Note that a small degree of hair cell recovery was observed in both regions after 56 days recovery. Labels: green = myosin VIIa (hair cells), red = phalloidin (actin filaments), blue = DAPI (cell nuclei)

I next characterized changes in junctional actin bands in response to ototoxic injury. Most prior studies of ototoxicity have focused on hair cell damage caused by treatment with aminoglycoside antibiotics or cisplatin. However, cisplatin causes damage to actin structures in the inner ear [16] and the vestibular organs of mice are relatively insensitive to aminoglycosides [17]. Instead, mouse vestibular hair cells were lesioned via systemic treatment with 3,3'-iminodipropanenitrile (IDPN) [18, 19]. Mice (2–4 months of age) received a single 4 mg/gm i.p. injection of IDPN and were allowed to recover for 3, 5, 7 or 56 days. Utricles were then removed, fixed and processed for histochemical labeling of hair cells and actin filaments. Images of utricles from IDPN-treated mice revealed a reduction in hair cell density in the central region of the utricle, when compared to undamaged controls (Fig. 1A-E). Hair cell density was quantified in 2,500 µm^2^ regions located within the striolar and extrastriolar regions of each utricle at 3, 5, 7 or 56 days after IDPN injection. These data revealed a progressive loss of hair cells in both regions over a seven day period. Consistent with a previous study [19], a modest hair cell recovery was observed in both regions after 56 days recovery (Fig. 1F, G, p < 0.0001).

**Fig. 2:**
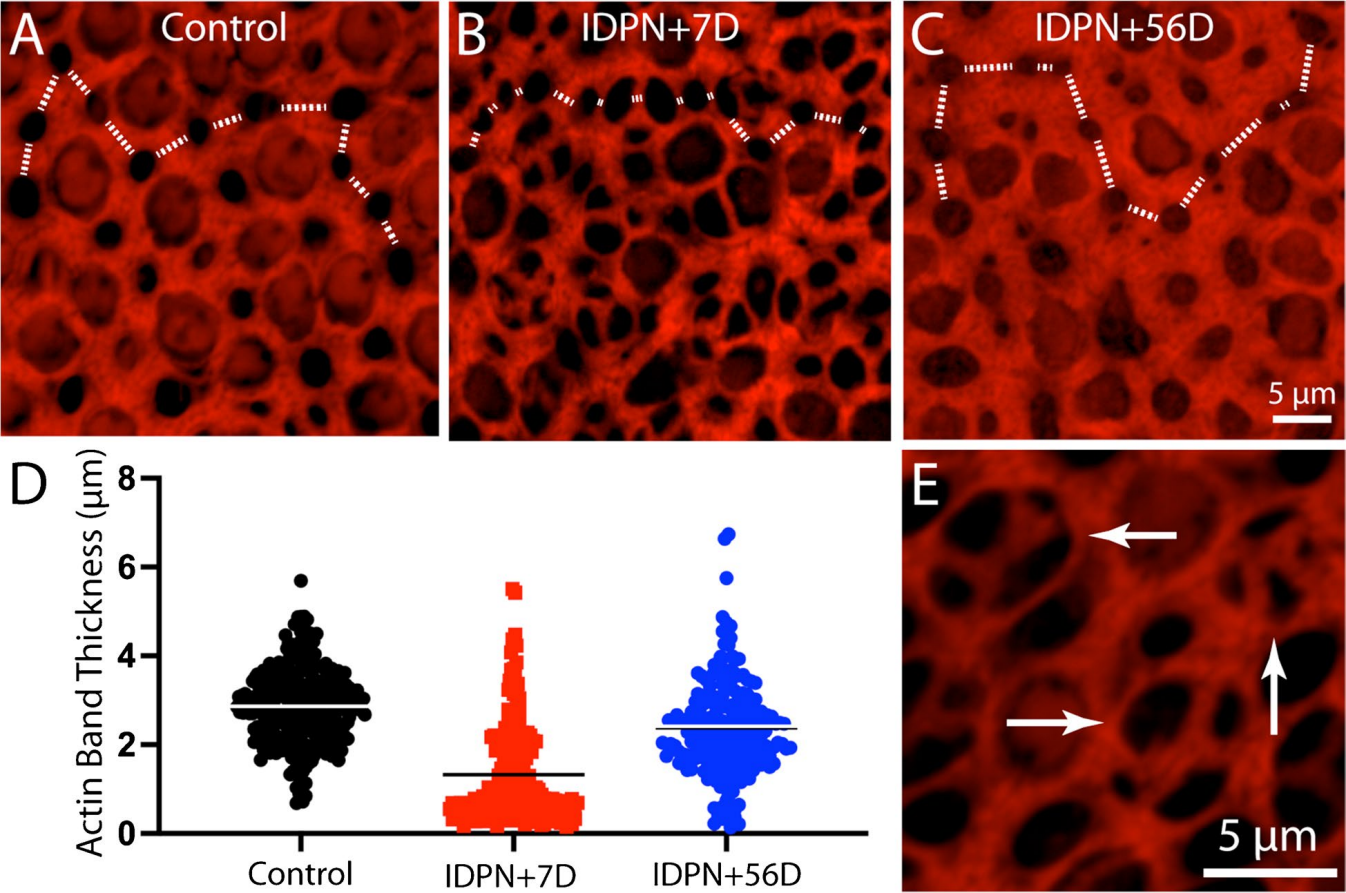
Changes in the thickness of junctional actin bands in the mouse utricle after IDPN-induced injury. The width of phalloidin-labeled junctional bands between adjoining supporting cells was quantified, moving from left to right across high magnification images (dotted white lines in **A**, **B**, **C**). (**D**): Quantitative data indicate that ototoxic injury resulted in a significant reduction in actin band thickness after seven days recovery. After 56 days recovery, the thickness of actin bands had nearly returned to control values. (**E**): Many of the thin actin bands in injured utricles form ‘rosettes or ‘scars’ that mediate resealing of the sensory epithelium (arrows). Color: red = phalloidin (actin filaments)

Having characterized IDPN-induced hair cell loss, I then examined resulting changes in the junctional actin belts. Ototoxic injury resulted in the formation of junctional actin bands with considerably reduced thickness, similar to the changes reported by Kaur et al. [20] following diphtheria toxin-induced hair cell loss in Pou4f3-huDTR transgenic mice. Utricles fixed at seven days after IDPN treatment had a mean junctional thickness of 1.20 ± 1.01 µm (n = 274 measurements from five specimens; Fig. 2B, D), Many of these junctions appeared to form ‘rosettes’ or ‘scars’ that were suggestive of epithelial repair after hair cell loss (Fig. 2E) [10]. Utricles fixed at 56 days post-IDPN had a mean junctional thickness of 3.22 ± 4.53 (n = 211 samples from five specimens; Fig. 2C, D). These data indicate that the acutely-injured utricle possesses many thin actin belts, but that normal junctional thickness is restored after sufficient recovery time.

**Fig. 3:**
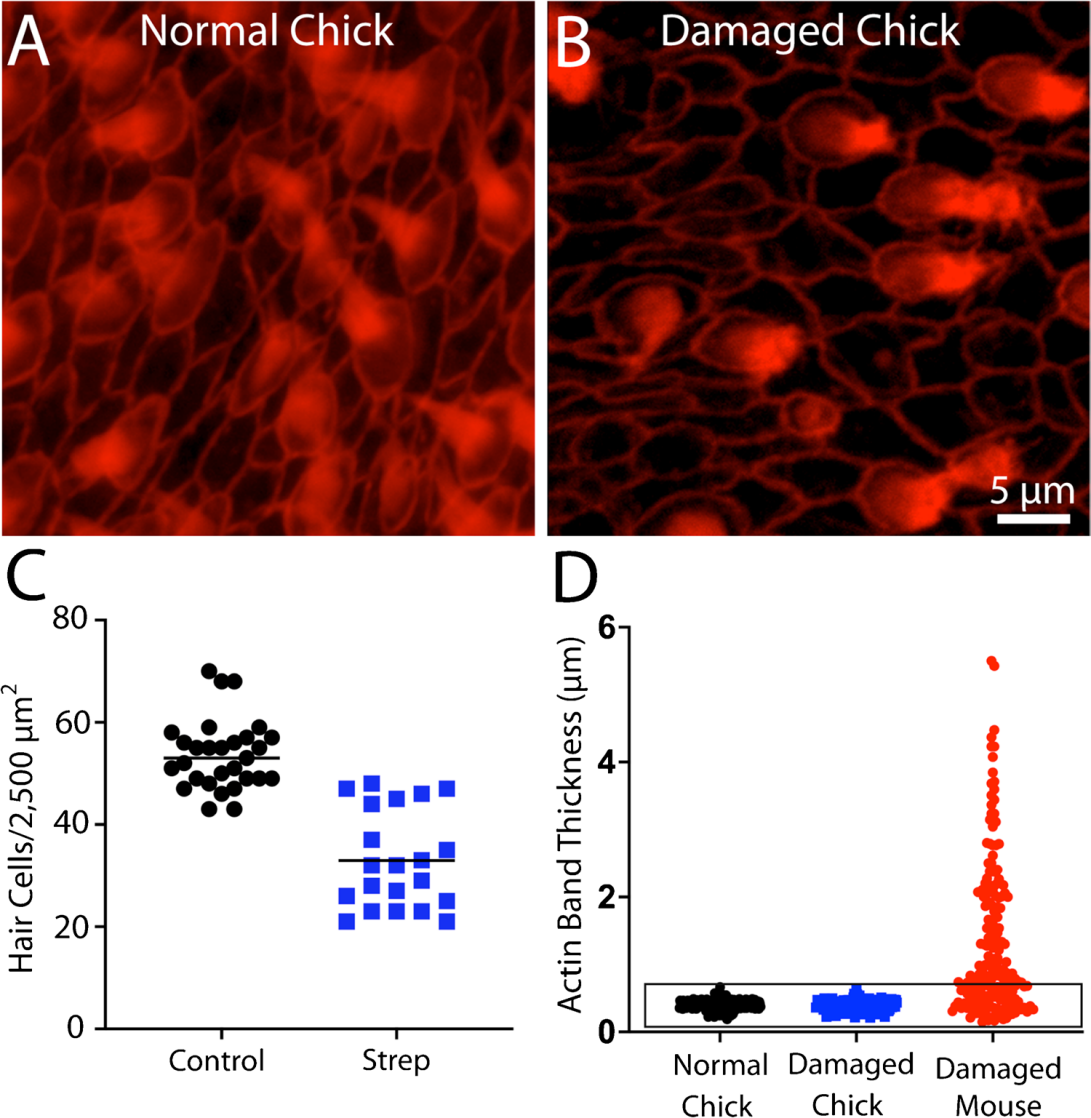
Ototoxic injury to the chick utricle does not affect the thickness of circumferential actin cables. Chicks received three daily infections of 1,200 mg/kg streptomycin sulfate and utricles were fixed 24 h after the final injection. (**A**, **B**): Images of phalloidin-labeled (red) cell–cell junctions in the normal and streptomycin-damaged chick utricle. (**C**): Streptomycin treatment caused ~ 40% reduction in hair cell density within the striolar region. (**D**): Quantification of actin band thickness from the striolar regions of normal and streptomycin-damaged utricles revealed no differences. Note, however, that potting data from the mouse utricle obtained at seven days after IDPN injection (shown in Fig. 2) on these same coordinates reveals that the thicknesses of many actin bands in the injured mouse utricle are comparable to those present in the chick utricle (D, data enclosed in box)

Since the dimensions of junctional actin bands may influence the regenerative properties of supporting cells, it was of interest to compare injury-evoked changes in actin bands in utricles of mice vs. chicks. Hair cell lesions in chicks (2–4 weeks post-hatch, n = 4) were created by giving three injections of 1,200 mg/kg streptomycin sulfate (one/day for three days) and then allowing them to recover for 24 h after the final injection. Fixed utricles were immunolabeled for myosin VIIa (to label hair cells) and phalloidin (to label actin junctions). Prior studies have shown that hair cell loss from this streptomycin regimen is limited to the striolar region of the utricle [14], so all data were obtained from the striola. Hair cell density in the striolar region of undamaged chick utricles was 53.6 ± 6.9 HCs/2,500 µm^2^ (n = 40 samples from five specimens), which decreased to 32.7 ± 9.6 HCs/2,000 µm^2^ after streptomycin treatment (n = 21 samples from eight specimens). The thickness of junctional actin bands in the utricles of streptomycin-treated chicks was 0.38 ± 07 µm (n = 202 measurements from eight specimens; Fig. 3B, C), which is nearly identical to the value reported above for uninjured utricles. Notably, plotting junctional thickness data obtained from injured mouse utricles on the same coordinates as data from chicks revealed that ototoxic injury caused many actin cables in the mouse utricle to acquire thicknesses comparable to those observed in the utricles of chicks (Fig. 3D).

**Fig. 4:**
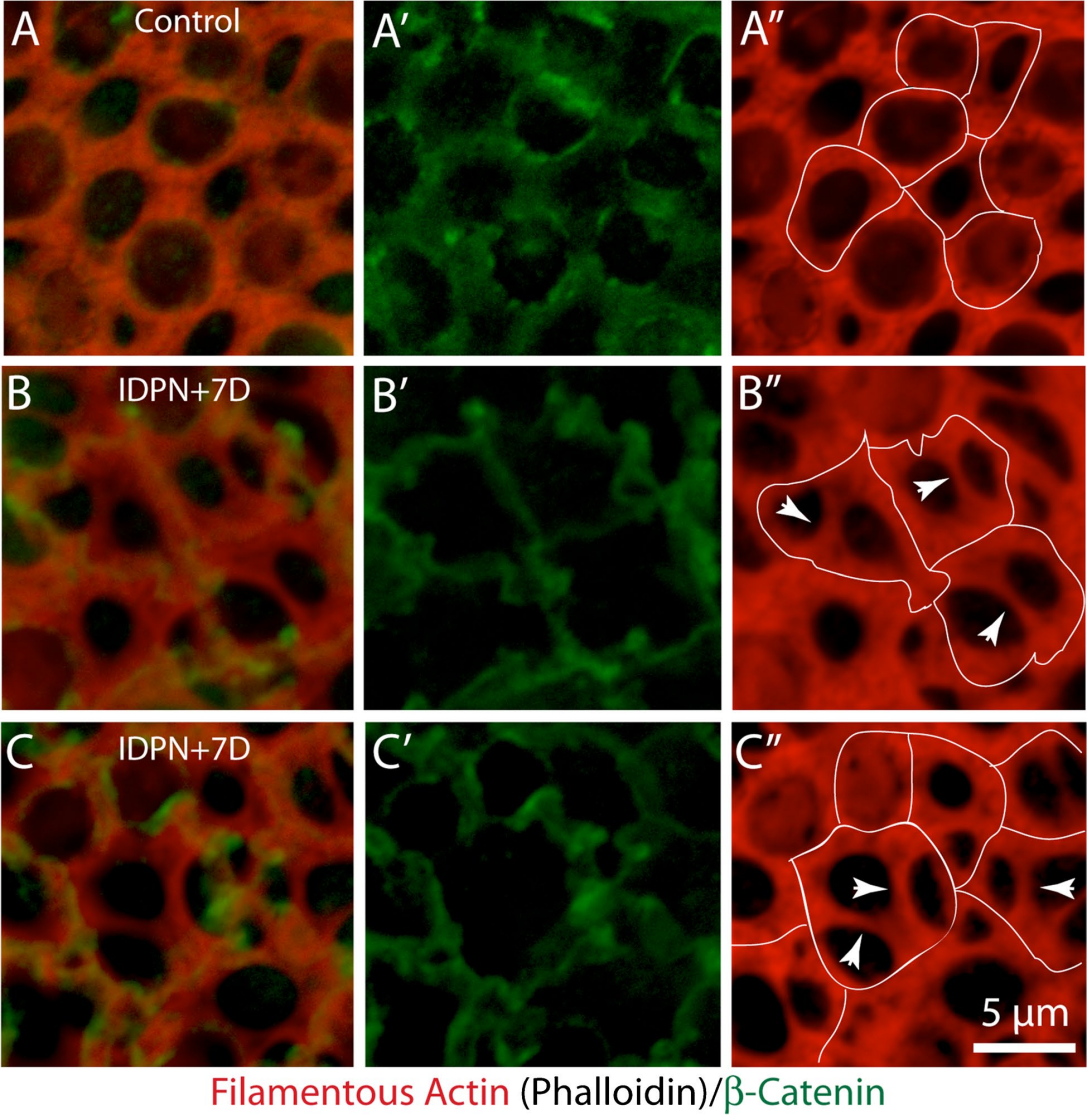
Identification of cell–cell junctions in the mouse utricle after ototoxic lesion. (**A**): In undamaged utricles, immunolabeling for β-catenin (**A**, **A**’, green) overlapped with junctional actin bands (phalloidin, red). Traces of β-catenin immunoreactivity (dotted lines) are shown in **A**’’. (**B**, **C**) Damaged utricles possessed both thick and thin actin bands, making it difficult to identify the sites of cellular junctions. Immunoreactivity for β-catenin (**B**’, **C**’ green) indicates that many actin bands are no longer associated with adherins junctions (**B**’’, **C**’’, arrowheads)

The loss of hair cells will create gaps in the lumen of the sensory epithelium, leading to disruption of the barrier between endolymph and perilymph. Such injury sites are quickly resealed by the formation of ‘scars’, which form when portions of 3–5 adjoining supporting cells extend into the epithelial space that was formally occupied by the lost hair cell [10]. In the present study, such structures were commonly observed in utricles fixed at 3–7 days after IDPN injection (e.g., Fig. 2E). They consisted of thin cables within the injured regions that were likely formed from actin processes that extended from supporting cells and joining together near the center of the lesion. This combination of thick and thin actin cables at the lumen made it difficult to definitively identify cell–cell junctions in surface views of the injured sensory epithelium. Instead, adherins junction were identified by immunoreactivity for β-catenin [21]. As expected, β-catenin labeling in undamaged utricles overlapped with the circumferential actin bands (Fig. 4A, A’, A’’). However, in IDPN-treated utricles, a subset of the thicker actin bands did not co-label for β-catenin (Fig. 4B, C), suggesting that they were no longer associated with cell–cell junctions.

**Fig. 5:**
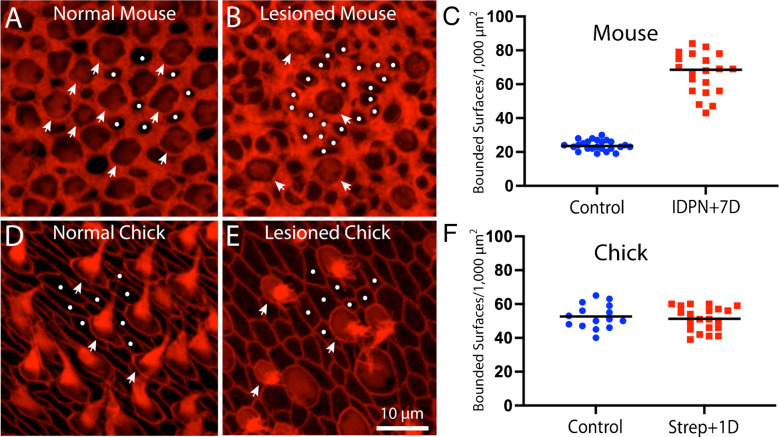
Quantification of F-actin-enclosed surfaces on the utricles of chicks and mice. (**A**): Surface view of an uninjured mouse utricle reveals regions that are enclosed by actin bands (phalloidin, red) and can be readily identified as either individual hair cells (arrowheads) or supporting cells (dots). (**B**): Similar view of a mouse utricle fixed at seven days after IDPN injection reveals many additional actin-bounded regions (dots), when compared with the undamaged utricle shown in (**A**). Also note the presence of fewer hair cells (arrowheads). (**C**): Quantification of actin-bounded regions without stereocilia or cuticular plates (e.g., dots in **A** and **B**) increased by ~ 3 × after IDPN ototoxicity. (**D**): Surface view of actin bands in an undamaged chick utricle also permits identification of hair cell and supporting cell surfaces. (**E**): Ototoxic injury to the chick utricle still permits identification of actin-bounded regions that are not hair cells. (**F**): Quantification of non-hair cell actin-bounded surfaces did not change after ototoxic injury, suggesting that the phalloidin-labeled junctional cables are still associated with cell–cell junctions

The number of supporting cells in whole-mounts of uninjured utricles can be determined by quantifying surfaces bounded by actin cables that do not possess features of hair cells (e.g., stereocilia bundles or myosin VIIa immunoreactivity). The structure of actin bands in chick utricles was not impacted by ototoxic lesion, suggesting that they remained associated with cell–cell junctions. However, the lumenal surfaces of injured mouse utricles possessed both actin bands that were associated with cell–cell junctions and numerous other actin bands that appeared to be confined to individual supporting cells and were no longer associated with cellular junctions. As a result, the borders of individual cells were difficult to distinguish in confocal surface views. To better clarify the relationship between enclosed actin regions and supporting cell numbers, I quantified the density of lumenal regions that were bounded by filamentous actin structures, but did not possess cuticular plates and/or stereocilia bundles (e.g., hair cells). The density of such regions in uninjured mouse utricles was 23.9 ± 3.0 regions/1,000 µm^2^ (n = 28 samples from five specimens), which likely reflects the density of supporting cells (Fig. 5A). However, the density of actin-bounded regions in utricles from IDPN-lesioned mice was 66.1 ± 12.1 regions/1,000 µm^2^ (Fig. 5B, C, n = 20 samples from five specimens). Since supporting cells of these utricles underwent little (if any) cell division after injury [19], the increase in actin-enclosed surfaces cannot be attributed to cellular addition. Instead, as noted above, many of the thicker actin cables no longer colocalized with cellular junctions, but remained present on the surfaces of individual supporting cells. The continued presence of these cables, when combined with the thinner actin cables that were formed to mediate epithelial repair, resulted in an increase in actin-bounded regions at the lumen of the epithelium (Fig. 5C). Thus, in acutely-injured utricles, it is no longer possible to quantify supporting cell density by simply counting actin-bounded regions. This observation also raises the possibltiy that the continued presence of thick actin cables within individual supporting cells (but no longer at cellular junctions) may affect the mechanical properties of those cells. In contrast, the density of actin-bound regions on the surfaces of chick utricles was not affected by ototoxic injury (Fig. 5D-F), suggesting that each actin-enclosed region still reflected an individual cell.

**Fig. 6:**
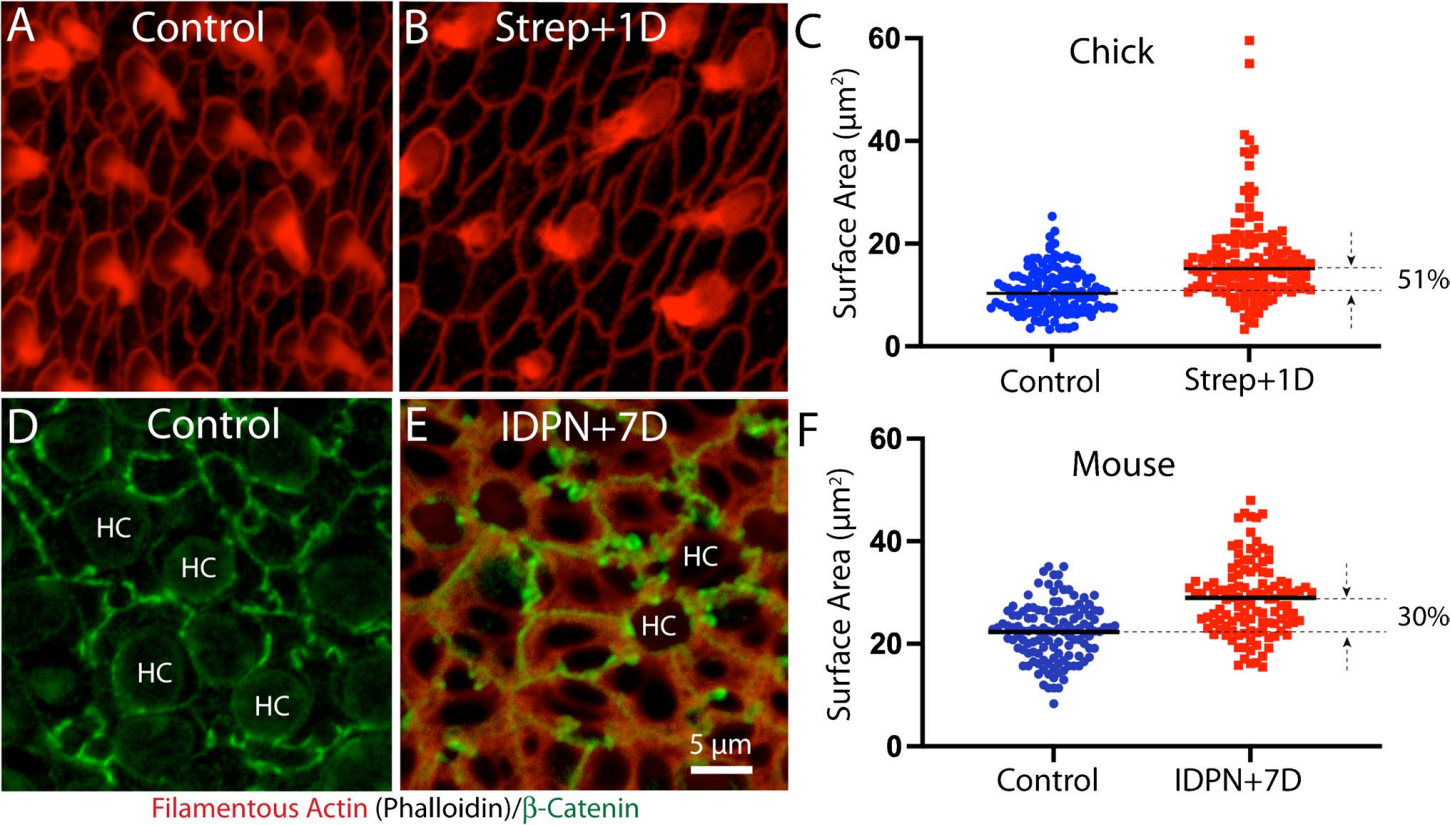
Quantification of the surface areas of supporting cells in normal and ototoxically-lesioned utricles. (**A**, **B**): Surface images of the chick utricle after labeling with phalloidin (red). (**C**): Although the number of supporting cell surfaces does not change after lesion (see Fig. 5), individual supporting cells have expanded their surfaces by ~ 50%, in order to reseal the epithelium. (**D**, **E**): Cell–cell junctions in the mouse utricle were labeled with β-catenin (green), which permitted quantification of cell surface area. (**F**): Resulting data indicate that loss of hair cells caused mouse supporting cells to expand by only ~ 30%, which was sufficient to repair the epithelium. The different relative expansion of chick vs. mouse supporting cells can be attributed to the differing numbers and sizes of these cells in both species (see text). *HC *hair cell

Repair of epithelial lesions will also cause expansion of the surfaces of supporting cells. Prior studies have shown that developmental growth and regeneration of the utricle is regulated by the Hippo/YAP signaling pathway [12–14], which can be triggered by cellular spreading [11]. In order to determine the extent of cellular expansion after hair cell loss, I next quantified the surface areas of supporting cells in normal and ototoxically-injured utricles of chicks and mice. Striolar supporting cells in uninjured chick utricles had a mean surface area of 10.8 ± 4.3 µm^2^ (n = 140 samples from 5 specimens), while those that had lost ~ 40% of their hair cells had a mean surface area of 16.8 ± 8.4 µm^2^ (n = 150 samples from 6 specimens; Fig. 6A-C). In contrast, supporting cells in the striolar region of uninjured mouse utricles had a mean surface area of 22.3 ± 5.5 µm^2^, while those that had lost ~ 60% of their hair cells after IDPN treatment had a mean surface area of 29.0 ± 7.6 µm^2^ (Fig. 6D-F; n = 126/117 samples from six specimens/group). These data reveal some important differences between supporting cells in the utricles of chicks and mice. First, supporting cells in uninjured mouse utricles have surface areas that are about twice as large as those in uninjured chick utricles (Fig. 6C, F). In addition, the loss of ~ 40% of hair cells from the chick utricle caused supporting cells to expand by ~ 50%, while a greater degree of hair cell loss in mouse utricles (~ 60%) led to expansion of supporting cell surfaces by only ~ 30%. In order rule out any possible influence of species-specific differences in the apical surfaces of hair cells, I quantified surface areas of hair cells in the striolar region of utricles from chicks and mice. Chick hair cells had a mean surface area of 16.7 ± 7.1 µm^2^ (n = 158 samples from five specimens), while mouse hair cells had a mean surface area of 17.1 ± 4.6 µm^2^ (n = 150 samples from five specimens). These data suggest that the differences in injury-evoked supporting cell expansion in chick vs. mouse cannot be attributed to differences in the surface areas of (lost) hair cells. In addition, hair cell density in uninjured utricles of mice and chicks is very similar (compare data in Figs. 1F and 3C). Together, these observations indicate that the surface areas of supporting cells in normal chick utricles are smaller than those in normal mouse utricles, and that chick supporting cells undergo a greater relative degree of cellular expansion in response to hair cell injury than do supporting cells of the mouse utricle.

## Discussion

### Link Between Actin Junctions, Cell Shape and Regenerative Proliferation

The vestibular organs of nonmammalian vertebrates are capable of regenerating hair cells after ototoxic injury [22]. Most replacement hair cells are produced by the proliferation of supporting cells, which divide to yield new hair cells and supporting cells. In contrast, hair cell regeneration in the vestibular organs of mammals is very limited and occurs by a process of transdifferentiation, whereby supporting cells change phenotype into replacement hair cells without first undergoing cell division [6]. Importantly, these newly-generated hair cells do not acquire mature physiological properties and do not restore proper vestibular function [3, 4]. Together, these observations suggest that the potential for regenerative cell proliferation in the inner ear is correlated with the ability for full recovery of sensory function.

The signals that regulate cell proliferation in the vertebrate inner ear are not fully understood, but a series of studies has suggested that the thickness of circumferential actin belts at cell–cell junctions may influence cell cycle entry [15]. Cellular junctions in the chick utricle possess thin actin cables, that permit significant cellular spreading and proliferation. In contrast, the junctional actin belts in the mouse utricle are considerably thicker than their chick counterparts and, during the development of the mouse utricle, the gradual thickening of cellular junctions is correlated with reduced spreading and proliferative ability [7, 9]. The thick circumferential actin bands of the mouse utricle also result in high epithelial stiffness, which may further limit cell cycle entry [23]. The present results demonstrate that ototoxic injury to the mouse utricle results in the formation numerous thin actin bands, many of which are of comparable thickness to those in the chick utricle (Fig. 3D). It is likely that many of these thin bands are created shortly after hair cell loss, in order to facilitate epithelial repair. It would be of interest to quantify the mechanical properties of acutely-injured vestibular epithelia of mice, to determine how the combination of thin and thick actin bands impacts overall epithelial stiffness.

**Fig. 7:**
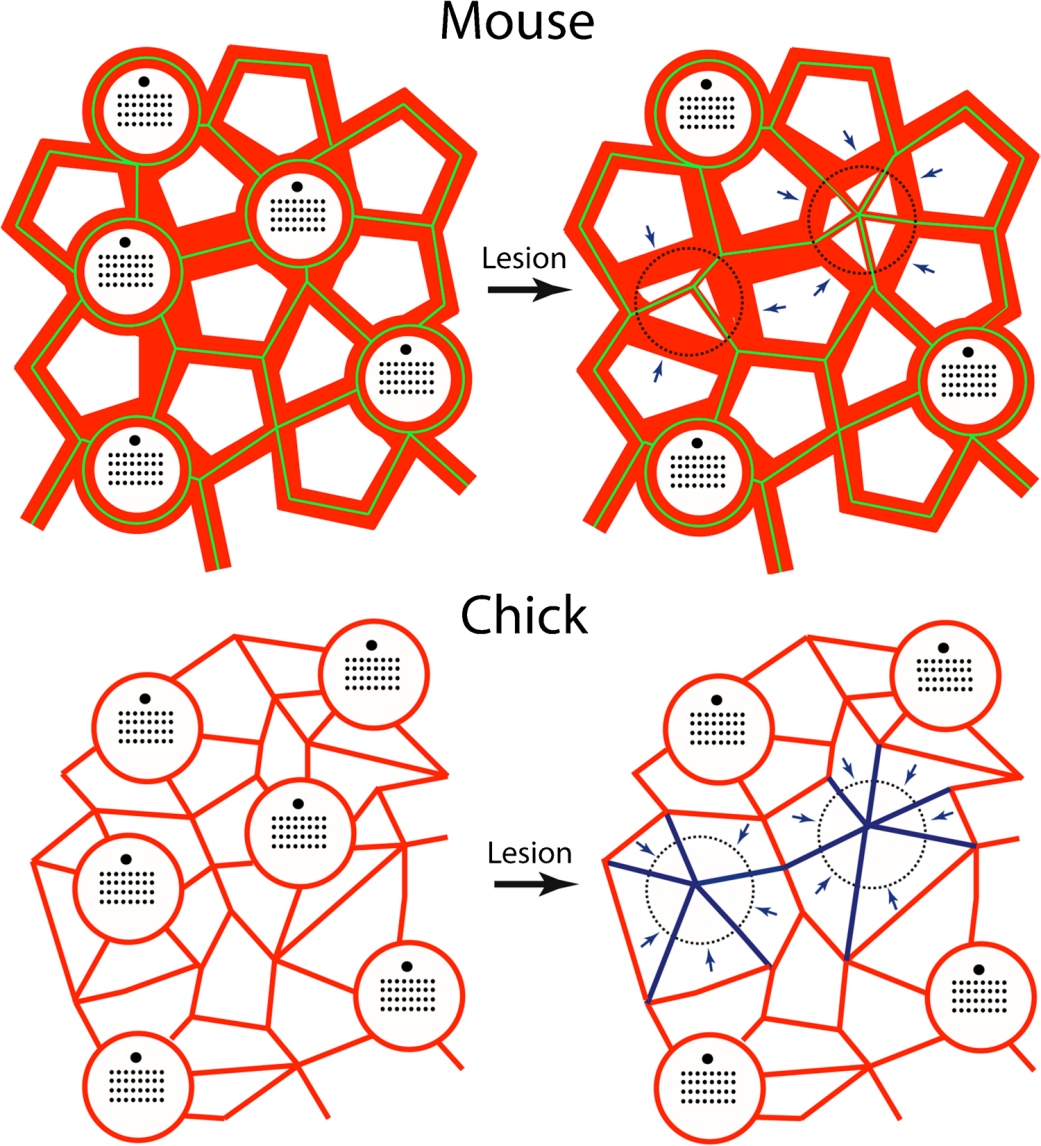
Mechanisms of epithelial repair in the utricles of mice and chicks. Junctional actin bands (red) in the mouse utricle are much thicker than those in the chick. Adherins junctions in the uninjured mouse utricle (green, based on β-catenin immunoreactivity-see Fig. 6) colocalize with these junctional bands. After hair cell loss, supporting cells extend thin actin bands to repair the epithelium and these also form new adherins junctions. Note, however, that many of the remaining thick bands no longer possess adherins junctions (arrows) and are likely to reside within individual supporting cells. Cell–cell junctions in the chick utricle contain thinner actin bands, which rearrange to reseal the sensory epithelium after hair cell injury (arrows, blue). Also note that the density of supporting cell surfaces in the undamaged chick utricle is about twice that of the mouse utricle. For this reason, hair cell loss causes the surface area of chick supporting cells to expand by a greater extent than in the mouse utricle

The YAP signaling pathway regulates cell proliferation in the utricles of chicks and mice [12–14], and data on injury-induced nuclear translocation of YAP1 in some of the chick utricles described in the present study has been published elsewhere [14]. Although the mechanisms responsible for YAP activation in the injured utricle are not completely understood, Hippo/YAP signaling can be activated via mechanical stretching [11, 24]. Activation of YAP signaling (as determined by nuclear translocation of the YAP1 protein) occurs in the injured chick utricle, but not in the injured mouse utricle [13, 14], so it is notable that the relative degree of injury-induced cellular expansion differs in the utricles of chicks vs. mice. Partial hair cell loss in the chick utricle (~ 40%) caused nearby supporting cells to expand their surfaces by about 50%, while a greater degree of hair cell loss in the mouse utricle (~ 60%) led to a ~ 30% expansion of supporting cells. This process is illustrated schematically in Fig. 7. In many cell types, mechanical stress (such as that caused by cellular expansion) inhibits Lats1/2, thereby preventing the degradation of YAP1 and allowing it to enter the nucleus and activate Hippo pathway genes [25]. The observation that treatment with a small molecule inhibitor of Lats kinases can induce nuclear translocation of YAP1 in supporting cells of the mouse utricle [26] suggests that signals upstream of Lats1/2 limit the proliferative ability of supporting cells. As such, the extent of expansion that occurs in mouse supporting cells after hair cell injury may not sufficient to inhibit Lats kinases, thereby preventing YAP1 translocation and regenerative proliferation. This prediction could be tested by culturing isolated sensory epithelia of mouse utricles on flexible substrates that permit experimentally-induced cellular stretching. Finally, mechanical regulation of cell proliferation can also involve the Src kinase signaling pathway [27], although the possible role of Src in otic regeneration remains to be studied.

What factors govern the extent of supporting cell expansion in response to hair cell loss? The present results indicate that the lumenal surface areas of supporting cells in the mouse utricle are about twice as large as in those in the chick utricle (see Fig. 6). The chicken utricle contains ~ 29,000 hair cells and the ratio of hair cells to supporting cells is estimated to be about 1:2.5 [28]. In contrast, the mouse utricle contains ~ 3,300 hair cells, with a hair cell to supporting cell ratio of 1:1.2 [29]. Since hair cell size and density are similar in both species, the larger surface areas of mouse supporting cells can be attributed to the fact that there are only about half as many supporting cells (per hair cell) in the mouse utricle vs. the chick utricle. Given the smaller surface areas of chick supporting cells, those cells will have to undergo a greater relative degree of expansion during epithelial repair, and this may account (in part) for differences in YAP activation and regenerative proliferation that are observed in the utricles of chicks vs. mice.

### Different Methods of Ototoxic Injury

One limitation of the present study is that different ototoxins were used to damage the ears of mice and chicks. These experiments were designed to compare injury response in the ears of both species following a moderate degree of hair cell loss, but there are currently no regimens that are known to reliably induce ototoxic injury in the ears of both mammals and birds. Vestibular hair cells in mature mice can be lesioned using Pou4f3-huDTR transgenic models [6, 20], but prior studies indicate that the mouse vestibular organs are relatively insensitive to aminoglycoside antibiotics (except when applied directly into the inner ear [30]). The vestibular organs of chicks can be readily lesioned by systemic treatment with various aminoglycosides [1, 14], but IDPN ototoxicity has not been characterized in nonmammalian species. The ototoxicity of IDPN has been well-studied in rodents [18, 19] and a significant hair cell lesion can be induced from a single systemic injection (e.g., Fig. 1). The extent of hair cell regeneration that is observed in mice after IDPN treatment is comparable to that observed after diphtheria toxin administration in the Pou4f3-huDTR model [6]. Since regenerated hair cells are produced by supporting cells, the fact that regeneration occurs after IDPN ototoxicity suggests that IDPN does not have any deleterious effects on mouse supporting cells. Similarly, we found that the utricles of both chicks and mice quickly resealed their epithelial barriers (via supporting cell extension), despite being treated with differing ototoxins. The similar extent of the ototoxic lesions (~ 60% in the mouse utricle, ~ 40% in the striola of the chick utricle), combined with rapid epithelial repair in both species, suggests that the injury responses were similar in both cases. I acknowledge, however, that a more optimal approach would involve the use of the same ototoxin (and at similar doses) in both chick and mouse. Finally, the data reported here were collected from mice of both sexes. No sex-related differences were observed, and data from both sexes were subsequently pooled together for analysis.

## Data Availability

Data reported in the manuscript will be made available upon reasonable request.
